# Association between Apgar scores of 7 to 9 and neonatal mortality and morbidity: population based cohort study of term infants in Sweden

**DOI:** 10.1136/bmj.l1656

**Published:** 2019-05-07

**Authors:** Neda Razaz, Sven Cnattingius, KS Joseph

**Affiliations:** 1 Division of Clinical Epidemiology, Department of Medicine Solna, Karolinska Institutet, Stockholm, Sweden; 2 Department of Obstetrics & Gynaecology, School of Population and Public Health, University of British Columbia and the Children’s and Women’s Hospital of British Columbia, Vancouver, BC, Canada

## Abstract

**Objective:**

To investigate associations between Apgar scores of 7, 8, and 9 (versus 10) at 1, 5, and 10 minutes, and neonatal mortality and morbidity.

**Design:**

Population based cohort study.

**Setting:**

Sweden.

**Participants:**

1 551 436 non-malformed live singleton infants, born at term (≥37 weeks’ gestation) between 1999 and 2016, with Apgar scores of ≥7 at 1, 5, and 10 minutes.

**Exposures:**

Infants with Apgar scores of 7, 8, and 9 at 1, 5, and 10 minutes were compared with those with an Apgar score of 10 at 1, 5, and 10 minutes, respectively.

**Main outcome measures:**

Neonatal mortality and morbidity, including neonatal infections, asphyxia related complications, respiratory distress, and neonatal hypoglycaemia. Adjusted odds ratios (aOR), adjusted rate differences (aRD), and 95% confidence intervals were estimated.

**Results:**

Compared with infants with an Apgar score of 10, aORs for neonatal mortality, neonatal infections, asphyxia related complications, respiratory distress, and neonatal hypoglycaemia were higher among infants with lower Apgar scores, especially at 5 and 10 minutes. For example, the aORs for respiratory distress for an Apgar score of 9 versus 10 were 2.0 (95% confidence interval 1.9 to 2.1) at 1 minute, 5.2 (5.1 to 5.4) at 5 minutes, and 12.4 (12.0 to 12.9) at 10 minutes. Compared with an Apgar score of 10 at 10 minutes, the aRD for respiratory distress was 9.5% (95% confidence interval 9.2% to 9.9%) for an Apgar score of 9 at 10 minutes, and 41.9% (37.7% to 46.4%) for an Apgar score of 7 at 10 minutes. A reduction in Apgar score from 10 at 5 minutes to 9 at 10 minutes was also associated with higher odds of neonatal morbidity, compared with a stable Apgar score of 10 at 5 and 10 minutes.

**Conclusions:**

In term non-malformed infants with Apgar scores within the normal range (7 to 10), risks of neonatal mortality and morbidity are higher among infants with lower Apgar score values, and also among those experiencing a reduction in score from 5 minutes to 10 minutes (compared with infants with stable Apgar scores of 10).

## Introduction

The most routinely used measure of health status of newborns is the Apgar score, typically quantified at 1, 5, and 10 minutes after birth.^1^ Our recent population based studies have shown that non-malformed term infants born with lower Apgar scores within the normal range (7 to 9) at 1, 5, or 10 minutes are at higher risk of adverse long term outcomes, such as epilepsy, cerebral palsy, having additional needs, and adverse child developmental health (compared with non-malformed term infants with an Apgar score of 10).^2^ ^3^ ^4^ Both the timing and the score are important: compared with an Apgar score of 10 at 5 and 10 minutes, an Apgar score of 9 at 5 minutes and an Apgar score of 9 at 10 minutes are both associated with an increased risk of cerebral palsy, with an Apgar score of 9 at 10 minutes conferring higher risk of cerebral palsy than an Apgar score of 9 at 5 minutes.^3^

The findings of differential risks associated with Apgar scores within the normal range are unexpected as it is commonly assumed that Apgar scores of 9 versus 10 are assigned arbitrarily. This belief is supported by international comparisons of Apgar scores, which show that the frequency of Apgar scores of 10 at 5 minutes vary from 8.8% in some countries to 92.7% in others.^5^ It is widely recognised that a low Apgar score, commonly defined as a score less than 7, is associated with increased risks of neonatal mortality,^6^ ^7^ morbidity,^8^ ^9^ ^10^ and long term outcomes^11^ ^12^ ^13^ ^14^ ^15^ ^16^; however, no previous study has investigated whether Apgar scores of 7, 8, and 9 are similarly associated with higher risks of neonatal mortality and morbidity. Quantifying associations between Apgar scores in the normal range and neonatal morbidity, such as neonatal infections, neonatal respiratory distress, and hypoxic-ischaemic encephalopathy is important because such conditions are known risk factors for later neurodevelopmental adversity in children. In this population based study of more than 1.5 million infants born in Sweden, we evaluated associations between Apgar scores of 7, 8, and 9 (versus 10) at 1, 5, or 10 minutes and risks of neonatal mortality and morbidity.

## Methods

We based our study on singleton live births in Sweden between 1999 and 2016, with data obtained from the Medical Birth Register.^17^ This database contains information on antenatal, obstetrical, and neonatal care that is prospectively recorded on standardised forms for more than 98% of births in Sweden. The most recent extensive validation of the Medical Birth Register showed that coverage and validity of most variables were high.^18^ Using the person-unique national registration numbers of mothers and infants, we linked data from the Birth Register to several national registries. The nationwide National Patient Register^19^ ^20^ includes diagnostic codes on hospital in-patient care since 1987 and hospital out-patient care from 2001. We coded diagnoses in the patient and birth registers using the Swedish versions of the International Classification of Diseases, 10th Revision (ICD-10) from 1997 onwards. We obtained information on neonatal deaths from the National Cause of Death Register, which includes information on all deaths in Sweden since 1961.^21^ Information on maternal education and country of origin was obtained from the Education Register and the Total Population Register, respectively.^22^ ^23^

### Study population

We analysed data for 18 years (1999-2016), during which 1 834 641 singleton live births were recorded in the Birth Register. We excluded preterm infants (≤36 completed weeks’ gestation, n=94 545), infants with major congenital malformations (n=60 762), and records with missing data on maternal or infant identification numbers (n=25 658), leaving 1 653 676 term (≥37 completed weeks’ gestation) singleton, non-malformed infants. Complete information on Apgar scores at 1 and 5 minutes was available for 1 645 396 infants (99%), of whom 1 620 473 (98.5%) also had information on Apgar scores at 10 minutes. We restricted our study population to infants with Apgar scores of 7 to 10 at 1, 5, and 10 minutes (n=1 551 436).

We obtained data on neonatal mortality and morbidity from nationwide Swedish registries: the Medical Birth Register and the Swedish patient and cause of death registers. Neonatal mortality was defined as infant deaths within the first 0-27 days after birth. Neonatal morbidity, assessed in the first 0-27 days after birth, included neonatal infections, asphyxia related neonatal complications (hypoxic-ischaemic encephalopathy and related conditions, and neonatal convulsions/seizures), neonatal hypoglycaemia, and respiratory distress (see supplementary table A for specific ICD-10 codes).

In Sweden, all women are offered an ultrasound scan at 18 weeks’ gestation or earlier for dating and screening for congenital abnormalities. In our study, we estimated gestational age (in completed weeks) using the following hierarchy: date of early second trimester ultrasonography (87.7%), date of last menstrual period (7.4%), or a postnatal assessment (4.9%).

Among maternal characteristics, we retrieved information on age at delivery, country of origin, highest attained level of education, cohabitation with a partner, parity, height, body mass index (BMI, kg/m^2^), and smoking during pregnancy. Maternal age at delivery was calculated as date of delivery minus the mother’s birth date, and parity was defined as the number of births to each mother (including the index birth). BMI was calculated using weight measured at registration to antenatal care (wearing light indoor clothing) and self reported height. BMI was categorised according to the World Health Organization groups as underweight (BMI <18.5), normal weight (18.5 to <25), overweight (25 to <30), obesity grade 1 (30 to <35), obesity grade 2 (35 to <40), or obesity grade 3 (≥40.0).^24^ We obtained information on cohabitation with a partner during the first antenatal visit. Mothers who reported daily smoking at the first antenatal visit and/or at 30 to 32 weeks’ gestation were classified as smokers, whereas mothers who stated that they were non-smokers were classified as such. Information on induction of labour and mode of delivery was noted on the obstetric record at onset of labour and after delivery, respectively.

### Statistical analyses

The frequency of each Apgar score value was calculated within categories of maternal and infant characteristics. Logistic regression was used to examine associations between Apgar scores of 7, 8, or 9 (versus 10) at 1, 5, or 10 minutes and neonatal mortality and each neonatal morbidity. Results were expressed as odds ratios with 95% confidence intervals. In the multivariable analyses, estimates were adjusted for maternal factors (age at childbirth, parity, country of birth, education, smoking, cohabitation with a partner, height and early pregnancy BMI) and birth characteristics of the infant (sex, gestational age in weeks, and year of birth). Lastly, the magnitude of absolute effects was quantified by calculating adjusted rate differences. The adjusted rate difference represents the number of excess cases of neonatal mortality and morbidity per 100 births among infants receiving an Apgar score of 7, 8, or 9 at 1, 5, and 10 minutes compared with infants receiving an Apgar score of 10. Two sided P values of less than 0.05 were considered to indicate statistical significance.

### Supplementary analyses

Pregnancy and delivery complications are associated with increased risks of a low Apgar score (0 to 6) and neonatal morbidity.^9^ ^10^ ^25^ We therefore also quantified the association between risk factors, such as gestational diabetes, pre-eclampsia, chorioamnionitis, placental abruption, premature rupture of membranes, induction of labour, mode of delivery, and meconium aspiration (see supplementary table A for specific ICD-10 codes) and Apgar scores of 7, 8, and 9 (versus 10) at 1, 5, or 10 minutes. Logistic regression with the Apgar score of interest (eg, 5 minute Apgar score of 9 versus 10) as the dependent variable was used to obtain odds ratios and 95% confidence intervals for each risk factor.

### Patient and public involvement

This study was based on analysis of information from linked databases and no patients were involved in designing the research question or the outcome measures, nor were they involved in developing plans for implementation of the study. No patients were asked to advise on interpretation or writing up of results.

## Results

Only 11% (163 800/1 551 436) of infants had an Apgar score of 10 at 1 minute, whereas 89% (1 373 314/1 551 436) and 97% (1 501 605/1 551 436) had a score of 10 at 5 and 10 minutes, respectively. Apgar scores of 10 at 5 minutes were less common in offspring of mothers who were primiparous, born in Sweden, shorter (<159 cm), or very obese (BMI ≥35 kg/m^2^), and less common in those who delivered at 37 weeks’ and ≥42 weeks’ gestation. The frequency of an Apgar score of 10 at 5 minutes was also lower in boys compared with girls (table 1).

**Table 1:** Maternal and birth characteristics according to Apgar score at 5 minutes: term singleton live births in Sweden, 1999 to 2016. Values are numbers (percentages) unless stated otherwise

Characteristics	Total No (n=1 551 436)	Apgar score at 5 mins			
		7 (n=3335)	8 (n=20 913)	9 (n=153 874)	10 (n=1 373 314)
Maternal age (years):					
≤19	23 127	50 (0.2)	282 (1.2)	2139 (9.2)	20 656 (89.3)
20-24	198 475	438 (0.2)	2714 (1.4)	18 913 (9.5)	176 410 (88.9)
25-29	475 860	1019 (0.2)	6516 (1.4)	47 785 (10.0)	420 540 (88.4)
30-34	537 904	1135 (0.2)	7034 (1.3)	53 528 (10.0)	476 207 (88.5)
≥35	316 070	693 (0.2)	4367 (1.4)	31 509 (10.0)	279 501 (88.4)
Parity:					
1	669 292	1951 (0.3)	12 270 (1.8)	70 176 (10.5)	584 895 (87.4)
2	579 715	907 (0.2)	5717 (1.0)	54 192 (9.3)	518 899 (89.5)
3	212 387	316 (0.1)	2081 (1.0)	20 614 (9.7)	189 376 (89.2)
≥4	90 042	161 (0.2)	845 (0.9)	8892 (9.9)	80 144 (89.0)
Country of birth:					
Sweden	1 208 381	2654 (0.2)	17 105 (1.4)	127 091 (10.5)	1 061 531 (87.8)
Other Nordic countries	25 012	52 (0.2)	344 (1.4)	2546 (10.2)	22 070 (88.2)
Non-Nordic countries	316 237	619 (0.2)	3452 (1.1)	24 109 (7.6)	288 057 (91.1)
Missing	1806	10 (0.6)	12 (0.7)	128 (7.1)	1656 (91.7)
Education (years):					
≤9	137 826	302 (0.2)	1699 (1.2)	12 315 (8.9)	123 510 (89.6)
10-11	186 936	400 (0.2)	2415 (1.3)	19 082 (10.2)	165 039 (88.3)
12	400 534	840 (0.2)	5556 (1.4)	40 138 (10.0)	354 000 (88.4)
13-14	212 304	454 (0.2)	2897 (1.4)	20 929 (9.9)	188 024 (88.6)
≥15	601 624	1299 (0.2)	8183 (1.4)	60 483 (10.1)	531 659 (88.4)
Missing	12 212	40 (0.3)	163 (1.3)	927 (7.6)	11 082 (90.7)
Smoking during pregnancy:					
No	137 0576	2947 (0.2)	18 678 (1.4)	136 846 (10.0)	1 212 105 (88.4)
Yes	120 616	264 (0.2)	1395 (1.2)	11 470 (9.5)	107 487 (89.1)
Missing	60 244	124 (0.2)	840 (1.4)	5558 (9.2)	53 722 (89.2)
Mother cohabits with partner:					
Yes	1 394 644	2978 (0.2)	18 683 (1.3)	139 471 (10.0)	1 233 512 (88.4)
No	85266	214 (0.3)	1262 (1.5)	8050 (9.4)	75 740 (88.8)
Missing	71 526	143 (0.2)	968 (1.4)	6353 (8.9)	64 062 (89.6)
Maternal height (cm):					
≤159	191 299	492 (0.3)	2938 (1.5)	19 241 (10.1)	168 628 (88.1)
160-164	371 064	883 (0.2)	5335 (1.4)	38 321 (10.3)	326 525 (88.0)
165-169	430 854	900 (0.2)	5846 (1.4)	43 272 (10.0)	380 836 (88.4)
≥170	480 148	903 (0.2)	5764 (1.2)	45 720 (9.5)	427 761 (89.1)
Missing	78 071	157 (0.2)	1030 (1.3)	7320 (9.4)	69 564 (89.1)
Maternal BMI:					
<18.5	33 673	42 (0.1)	350 (1.0)	2608 (7.7)	30 673 (91.1)
18.5-24.9	857 501	1674 (0.2)	10 666 (1.2)	81 478 (9.5)	763 683 (89.1)
25-29.9	356 678	880 (0.2)	5123 (1.4)	37 291 (10.5)	313 384 (87.9)
30-34.9	116 886	290 (0.2)	1925 (1.6)	12 973 (11.1)	101 698 (87.0)
≥35	47 352	150 (0.3)	901 (1.9)	5605 (11.8)	40 696 (85.9)
Missing	139 346	299 (0.2)	1948 (1.4)	13 919 (10.0)	123 180 (88.4)
Infant sex:					
Boy	784 782	1785 (0.2)	11 461 (1.5)	84 045 (10.7)	687 491 (87.6)
Girl	766 654	1550 (0.2)	9452 (1.2)	69 829 (9.1)	685 823 (89.5)
Gestational age (weeks):					
37	33 932	136 (0.4)	709 (2.1)	3798 (11.2)	29 289 (86.3)
38	130 721	372 (0.3)	1996 (1.5)	12 862 (9.8)	115 491 (88.3)
39	279 225	528 (0.2)	3424 (1.2)	25 449 (9.1)	249 824 (89.5)
40	410 458	766 (0.2)	4669 (1.1)	38 540 (9.4)	366 483 (89.3)
41	387 388	747 (0.2)	5072 (1.3)	38 661 (10.0)	342 908 (88.5)
≥42	309 712	786 (0.3)	5043 (1.6)	34 564 (11.2)	269 319 (87.0)
Year of delivery:					
1999-2004	447 250	794 (0.2)	6554 (1.5)	51 738 (11.6)	388 164 (86.8)
2005-08	347 334	617 (0.2)	4702 (1.4)	34 959 (10.1)	307 056 (88.4)
2009-12	373 705	814 (0.2)	4640 (1.2)	33 154 (8.9)	335 097 (89.7)
2013-16	383 147	1110 (0.3)	5017 (1.3)	34 023 (8.9)	342 997 (89.5)

### Neonatal mortality and morbidity

**Fig 1:**
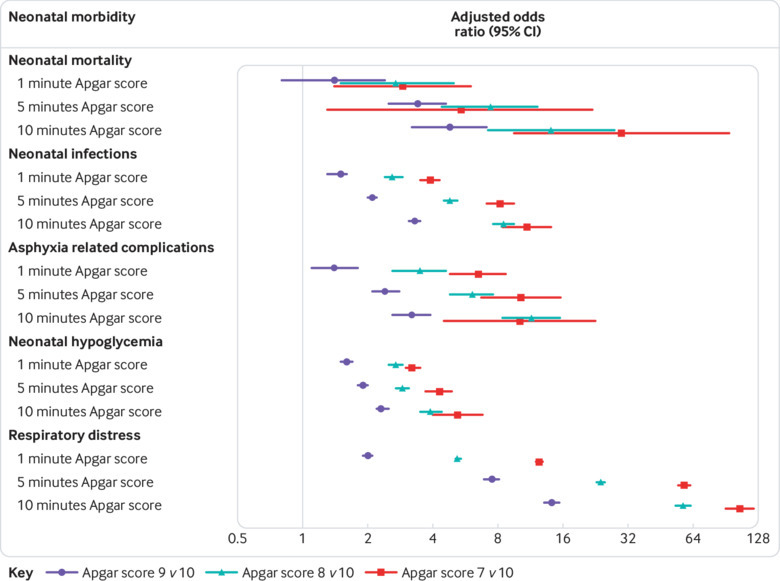
Associations between Apgar scores of 7, 8, and 9 at 1, 5, and 10 minutes and neonatal mortality and morbidity among term singleton live births in Sweden, 1999˗2016

Apgar scores of 7, 8, and 9 at 1, 5, and 10 minutes were strongly associated with neonatal mortality and morbidity, compared with an Apgar score of 10 at 1, 5, and 10 minutes (fig 1, supplementary table B). Compared with an Apgar score of 10 at 10 minutes, adjusted odds ratios for neonatal mortality increased from 4.8 for an Apgar score of 9 at 10 minutes to 29.8 for an Apgar score of 7 at 10 minutes (fig 1; supplementary table B). Furthermore, adjusted odds ratios between lower Apgar score values and neonatal mortality and each neonatal morbidity were higher with increasing time after birth. For example, compared with an Apgar score of 10 at 1 minute, an Apgar score of 9 at 1 minute was associated with 1.5-fold higher adjusted odds of neonatal infections, whereas the association was larger at 5 and 10 minutes (adjusted odds ratios 2.1 and 3.3, respectively). Asphyxia related complications, neonatal hypoglycaemia, and respiratory distress were also strongly associated with Apgar scores of 7, 8, and 9, and adjusted odds ratios increased with time since birth. The adjusted rate difference for respiratory distress was 9.5% (95% confidence interval 9.2% to 9.9%) for an Apgar score of 9 at 10 minutes and increased to 41.9% (37.7% to 46.4%) for an Apgar score of 7 at 10 minutes, compared with an Apgar score of 10 at 10 minutes (table 2). Lastly, the association between Apgar score and neonatal morbidity remained strong regardless of mode of delivery, and the highest odds ratios were observed for infants born following a non-instrumental vaginal delivery (supplementary table C).

**Table 2:** Adjusted rate differences (per 100 births) for neonatal mortality and morbidity outcomes for Apgar scores of 7, 8, and 9 at 1, 5, and 10 minutes compared with a score of 10, in Sweden, 1999 to 2016

Variables	1 min Apgar score			5 min Apgar score			10 min Apgar score	
	No (%)	Adjusted RD (95% CI)*		No (%)	Adjusted rate difference (95% CI)*		No (%)	Adjusted rate difference (95% CI)*
**Neonatal mortality**								
Apgar scores:								
7	16 (0.03)	0.02 (0.004 to 0.1)		4 (0.1)	0.04 (0.003 to 0.2)		5 (0.6)	0.6 (0.2 to 1.9)
8	42 (0.03)	0.02 (0.005 to 0.04)		22 (0.1)	0.1 (0.03 to 0.1)		11 (0.2)	0.3 (0.1 to 0.5)
9	207 (0.02)	0.004 (−0.002 to 0.01)		67 (0.04)	0.02 (0.02 to 0.04)		35 (0.1)	0.1 (0.04 to 0.1)
10	17 (0.01)	Reference		189 (0.01)	Reference		231 (0.02)	Reference
**Neonatal infections**								
Apgar scores:								
7	1252 (2.4)	1.5 (1.3 to 1.7)		210 (6.3)	5.5 (4.7 to 6.5)		71 (8.7)	8.3 (6.2 to 11)
8	2051 (1.7)	0.8 (0.7 to 1)		856 (4.1)	2.9 (2.7 to 3.2)		375 (7.4)	6.3 (5.5 to 7.1)
9	9834 (0.8)	0.3 (0.2 to 0.3)		2595 (1.7)	0.8 (0.8 to 0.9)		1288 (2.9)	1.9 (1.8 to 2.1)
10	823 (0.5)	Reference		10 299 (0.7)	Reference		12 226 (0.8)	Reference
**Asphyxia related complications**								
Apgar scores:								
7	178 (0.3)	0.2 (0.2 to 0.3)		25 (0.7)	0.6 (0.4 to 1.0)		6 (0.7)	0.7 (0.3 to 1.7)
8	218 (0.2)	0.1 (0.1 to 0.1)		105 (0.5)	0.4 (0.3 to 0.5)		54 (1.1)	0.8 (0.6 to 1.2)
9	834 (0.1)	0.02 (0.01 to 0.03)		259 (0.2)	0.1 (0.1 to 0.1)		116 (0.3)	0.2 (0.1 to 0.2)
10	69 (0.04)	Reference		910 (0.1)	Reference		1123 (0.1)	Reference
**Neonatal hypoglycaemia**								
Apgar scores:								
7	1799 (3.5)	2.4 (2.2 to 2.8)		228 (6.8)	5.4 (4.4 to 6.4)		74 (9.1)	7.4 (5.3 to 10.2)
8	3539 (2.9)	1.9 (1.7 to 2.1)		1038 (5.0)	3.1 (2.8 to 3.4)		354 (7.0)	5.1 (4.4 to 6.0)
9	18 815 (1.6)	0.7 (0.6 to 0.8)		4496 (2.9)	1.5 (1.3 to 1.6)		1765 (4.0)	2.3 (2.1 to 2.6)
10	1460 (0.9)	Reference		19 851 (1.4)	Reference		23 420 (1.6)	Reference
**Respiratory distress**								
Apgar scores:								
7	3736 (7.3)	6.0 (5.6 to 6.6)		1127 (33.8)	27.9 (26.1 to 29.9)		431 (52.8)	41.9 (37.7 to 46.4)
8	4920 (4.0)	3.1 (2.9 to 3.4)		3628 (16.3)	14.6 (14 to 15.2)		1952 (38.5)	31.9 (30.2 to 33.5)
9	12 732 (1.0)	0.5 (0.4 to 0.6)		6446 (4.2)	3.2 (3 to 3.3)		5101 (11.6)	9.5 (9.2 to 9.9)
10	896 (0.5)	Reference		11 083 (0.8)	Reference		14 800 (1.0)	Reference

* Adjusted for maternal factors (age at childbirth, parity, country of birth, education, smoking, cohabitation with a partner, height, early pregnancy BMI) and birth characteristics of the infant (sex, gestational age in weeks, year of birth).

### Combinations

Table 3 shows adjusted odds ratios for neonatal mortality and morbidity in relation to changes in Apgar score values from 5 to 10 minutes. A reduction of Apgar score from 10 at 5 minutes to 9 at 10 minutes was associated with higher adjusted odds ratios for neonatal infections, neonatal hypoglycaemia, and respiratory distress (compared with an Apgar score of 10 at both 5 and 10 minutes). For instance, compared with Apgar scores of 10 at both time points, a reduction in Apgar score from 10 at 5 minutes to 9 at 10 minutes was associated with a 4.1-fold higher odds of neonatal infections. Compared with Apgar scores of 10 at both time points, infants whose Apgar scores increased from 9 at 5 minutes to 10 at 10 minutes also had higher relative odds for all outcomes including neonatal mortality. For example, compared with Apgar scores of 10 at both time points, an improvement from an Apgar score of 9 at 5 minutes to 10 at 10 minutes was associated with a 1.8-fold higher odds of neonatal infections (adjusted odds ratio 1.8, 95% confidence interval 1.7 to 1.9).

**Table 3:** Combinations of Apgar scores at 5 and 10 minutes and adjusted odds ratios for neonatal mortality and morbidity among term singleton live births in Sweden, 1999˗2016

Morbidity	Apgar score		No (%)	Adjusted odds ratio (95% CI)*
	5 min	10 min		
Neonatal mortality	10	10	188 (0.01)	Reference
	10	9	1 (0.1)	-
	9	10	39 (0.03)	2.8 (2.0 to 4.0)
	9	9	27 (0.1)	5.5 (3.5 to 8.7)
	9	<9	1 (0.2)	-
Neonatal infections	10	10	10 266 (0.7)	Reference
	10	9	27 (3.7)	4.1 (2.7 to 6.4)
	9	10	1716 (1.4)	1.8 (1.7 to 1.9)
	9	9	848 (2.5)	3.0 (2.8 to 3.3)
	9	<9	31 (6.0)	6.9 (4.6 to 10.4)
Asphyxia related complications	10	10	907 (0.1)	Reference
	10	9	2 (0.3)	-
	9	10	194 (0.2)	2.4 (2.0 to 2.8)
	9	9	63 (0.2)	2.6 (2.0 to 3.5)
	9	<9	2 (0.4)	-
Neonatal hypoglycaemia	10	10	19 814 (1.4)	Reference
	10	9	31 (4.3)	2.5 (1.7 to 3.7)
	9	10	3225 (2.7)	1.8 (1.7 to 1.8)
	9	9	1239 (3.6)	2.3 (2.2 to 2.4)
	9	<9	32 (6.2)	3.8 (2.6 to 5.6)
Respiratory distress	10	10	10 883 (0.8)	Reference
	10	9	156 (21.6)	29.0 (23.9 to 35.3)
	9	10	3138 (2.6)	3.3 (3.2 to 3.4)
	9	9	3088 (9.0)	11.7 (11.1 to 12.2)
	9	<9	220 (42.6)	77.1 (63.8 to 93.3)

* Adjusted for maternal factors (age at childbirth, parity, country of birth, education, smoking, cohabitation with a partner, height and early pregnancy BMI) and birth characteristics of the infant (sex, gestational age in weeks, and year of birth).

### Supplementary analyses

Pregnancy and delivery factors, including gestational diabetes, pre-eclampsia, chorioamnionitis, placental abruption, induced onset of labour, vaginal instrumental or caesarean delivery, and meconium aspiration were associated with Apgar scores of 7, 8, and 9 (versus 10) at 5 and 10 minutes (see supplementary table D). The strength of association differed markedly, and the highest relative odds were obtained for pre-eclampsia, chorioamnionitis, placental abruption, vaginal instrumental delivery, emergency caesarean delivery, and meconium aspiration. Premature rupture of the membranes was not associated with increased odds for Apgar scores of 7, 8, or 9 at 5 or 10 minutes. Pregnancy and delivery factors were not or were only modestly associated with Apgar scores of 7, 8, or 9 versus 10 at 1 minute, except for vaginal instrumental delivery and meconium aspiration, which were associated with markedly higher odds of an Apgar score of 7, 8, or 9 at 1 minute (eg, the adjusted odds ratio for an Apgar score of 9 versus 10 at 1 minute for meconium aspiration was 3.7 (95% confidence interval 1.9 to 7.2)).

## Discussion

In this nationwide Swedish cohort study, we found that a lower Apgar score within the normal range at 1, 5, and 10 minutes is strongly associated with increased risks of neonatal mortality and morbidity. Furthermore, we found progressively higher relative odds of neonatal mortality, infections, asphyxia related complications, neonatal hypoglycaemia, and respiratory distress with lower Apgar scores (7 to 9) at 1, 5, and 10 minutes. The relative odds of neonatal mortality and morbidity associated with lower Apgar scores (in the normal range) increased with increasing time from birth. A small change in Apgar score from 5 minutes to 10 minutes (eg, from 10 to 9) was also associated with an increased risk of neonatal morbidity. Pregnancy and delivery related factors, such as pre-eclampsia, chorioamnionitis, placental abruption, induced onset of labour, vaginal instrumental delivery, and meconium aspiration were associated with Apgar scores of 7 to 9 (versus 10), suggesting that low Apgar scores in the normal range represent early prognostic indicators highlighting the effects of pregnancy and delivery complications on neonatal morbidity.

### Strengths and weaknesses of this study

Our study included more than 1.5 million births. We included all eligible births in Sweden over an 18 year period, thereby avoiding selection bias. Furthermore, we were able to adjust for several important confounders in multivariable analyses. In Sweden, all citizens have free access to uniform publicly funded healthcare, which contributes to high internal validity. However, the Apgar score is not subject to quality control measures and is prone to interobserver variability,^26^ and there are known international differences in the frequency of scores within the normal range.^27^ Nevertheless, the Apgar score has been shown to have good internal validity and could provide useful information about national trends in newborn health.^5^ Lastly, we lacked information about neonatal interventions and umbilical cord blood gases analysis, which could influence Apgar scores and neonatal mortality and morbidity. Future studies should examine the relation between acidosis and Apgar score within the normal ranges and its impact on neonatal morbidity.

### Comparison with other studies

Previous studies have shown that an Apgar score of less than 7 is associated with neonatal morbidity, including meconium aspiration, neonatal respiratory distress, hypoxic-ischaemic encephalopathy, and infant mortality.^6^ ^9^ ^10^ ^25^ The relation between Apgar score of less than 7 and neonatal and infant mortality is mainly attributed to anoxia or infections.^6^ Our study expands on these findings by showing that even “normal” Apgar scores (7 to 9) are strongly associated with higher risks of neonatal mortality and neonatal morbidity, and neonatal morbidity is associated with risks of long term neurological disorders.^28^ These findings are consistent with evidence suggesting that some infants with reassuring Apgar scores (7 to 9) with acidaemia have higher rates of adverse outcomes.^29^ Furthermore, risks associated with a low Apgar score (in the normal range) at 10 minutes were generally higher than those associated with the same score at 1 or 5 minutes. The strong relations between Apgar scores of 7, 8, and 9 and neonatal morbidity, and the associations between pregnancy complications and lower Apgar scores in the normal range observed in our study, provide insight into previous findings of increased risks of cerebral palsy, epilepsy, autism, and adverse developmental outcomes in children with Apgar scores of 7, 8, and even 9, compared with an Apgar score of 10.^2^ ^3^ ^30^

In our study only 11% of infants had an Apgar score of 10 at 1 minute, which is typically attributable to a reduction in score for skin colour. This finding warrants attention as our results show that an Apgar score of 9 at 1 minute was associated with higher risk of neonatal morbidity. Although a reduction in the 5 minute Apgar score due to colour is independently associated with an increased risk of infant mortality,^8^ current guidelines consider Apgar scores of 7 or more at 1 and 5 minutes to be reassuring.^31^ Our findings of an incremental increase in the risks of neonatal mortality and morbidity among infants with Apgar scores of 7, 8, and 9 at 1, 5, and 10 minutes, along with previous results of a linear relation^2^ ^3^ ^4^ ^30^ between decreasing Apgar scores and increasing risk of adverse neurodevelopmental outcomes, suggest that efforts should be made to reduce the rate of low Apgar scores within the normal range and to strive for an Apgar score of 10 immediately after birth.

Seizures, intracranial haemorrhage, and birth asphyxia have been shown to be associated with Apgar scores of less than 7 at 5 and 10 minutes.^9^ Our study is, to our knowledge, the first to show an association between a reduction in Apgar score (from 10 at 5 minutes to ≤9 at 10 minutes) and increased risks of neonatal mortality and morbidity, including infections, asphyxia related conditions, and respiratory distress. A low Apgar score at 5 minutes and 10 minutes might indicate the lack of an optimal response to resuscitation and could imply an adverse long term prognosis.^9^ A reduction in Apgar score from 5 minutes to 10 minutes is also associated with higher risks of later developing cerebral palsy, epilepsy, or other developmental adversity.^3^ ^4^ Although Apgar scores at 10 minutes are often not recorded in the medical charts if scores are within the normal range (7 to 10) at 5 minutes, our findings suggest that all newborns should be assigned an Apgar score at 10 minutes, regardless of their score at 1 minute and 5 minutes. This will enable at-risk neonates to be identified and monitored to minimise the risk of adverse outcomes.

### Conclusions

Our study shows that low Apgar scores within the normal range (7 to 10) are strongly associated with neonatal mortality and morbidity and that these associations are substantially stronger with increasing time after birth. Additionally, a decrease in Apgar score from 10 at 5 minutes to 9 or less at 10 minutes is associated with a higher risk of neonatal morbidity. Our findings provide strong evidence to support the proposition that the optimal Apgar score is 10 at each time point, and all newborns should be assigned an Apgar score at 10 minutes, regardless of their score at 1 minute and 5 minutes.

**What is already known on this topic**

An Apgar score of less than 7 has implications for neonatal mortality, morbidity, and long term neurodevelopmental outcomes
No previous study has investigated whether Apgar scores of 7, 8, and 9 (compared with 10) are associated with neonatal mortality and morbidity

**What this study adds**

In term, non-malformed infants, the risks of neonatal mortality and morbidity were higher among those with lower Apgar score values within the normal range (7 to 9) at 1, 5, and 10 minutes
Even a small change in Apgar score from 5 minutes to 10 minutes was associated with higher risks of neonatal morbidity
The optimal Apgar score is 10 at each time point, and all newborns should be assigned an Apgar score at 10 minutes, regardless of their score at 1 minute and 5 minutes

## Web extra

Extra material supplied by authors

## Notes

Contributors: NR and SC had full access to all the data in the study and take full responsibility for the integrity of the data and the accuracy of the data analysis. They are the guarantors. NR, SC, and KSJ conceived and designed the study. All authors interpreted the data and critically revised the manuscript for important intellectual content. NR drafted the manuscript and carried out the statistical analysis. SC obtained funding and provided administrative, technical, or material support.

Ethical approval: This study was approved by the regional ethic review board at Karolinska Institutet, Stockholm, Sweden (No 2018/1377-32).

Data sharing: No additional data available.

Transparency: The lead author (NR) affirms that the manuscript is an honest, accurate, and transparent account of the study being reported; that no important aspects of the study have been omitted; and that any discrepancies from the study as planned (and, if relevant, registered) have been explained.
