# Supplementary material for: Association between Apgar scores of 7 to 9 and neonatal mortality and morbidity: population based cohort study of term infants in Sweden
Source: BMJ. 2019 May 7;365:l1656. doi: 10.1136/bmj.l1656 (PMC6503461; doi:10.1136/bmj.l1656)
Supplement: Supplementary file 1 — Supplementary materials: Supplementary tables A-D [file razn048528.ww1.docx]

**Supplementary Web Appendix**

This appendix has been provided by the authors to give the readers additional information about their work.

| **Table of Contents** |
| --- |
| Table A. ICD-10 codes for maternal and neonatal diseases. |
| Table B. Crude and adjusted odds ratios of neonatal mortality and morbidity for Apgar score of 7, 8 and 9 at 1, 5, and 10 minutes among term, singleton live births in Sweden, 1999˗2016 |
| Table C. Association between Apgar scores of 7, 8 or 9 at 1, 5 and 10 minutes and neonatal morbidity by mode of delivery among term, singleton live births in Sweden, 1999˗2016  Table D. Associations between pregnancy and delivery factors and Apgar scores of 7, 8 or 9 at 1, 5 and 10 minutes among term, singleton live births in Sweden, 1999˗2016 |

**Supplementary Table A.** **ICD-10 codes for maternal and neonatal diseases.**

| **Diseases** | **ICD-10 codes** |
| --- | --- |
| **Risk Factors**** |  |
| Placental abruption | ICD-10: O45 |
| Premature rupture of membranes | ICD-10: O42 |
| Preeclampsia and eclampsia | ICD-10: O14 and O15 |
| Gestational diabetes | ICD-10: O24.4 |
| Chorioamnionitis | ICD-10: O411 |
| **Neonatal Morbidity ^‡^** |  |
| Neonatal infections | ICD-10: P35 to P39 |
| Meconium aspiration | ICD-10: P24.0 |
| Convulsions/seizures | ICD-10: P90 |
| Hypoglycemia | ICD-10: P70 |
| Respiratory distress | ICD-10: P22 |
|  |  |
|  |  |

Diseases were defined using the Swedish version of the International Classification of Diseases, tenth revision (ICD-10).

^‡^ Neonatal morbidity and risk factors were identified from ICD-10 codes from the Medical Birth Register and the National Patient Register.

^ǁ^ICD-10 codes of minor malformations are available at <http://www.socialstyrelsen.se/register/halsodataregister/medicinskafodelseregistret/Documents/diagnoser->s

om-inte-ska-rapporteras.pdf

| **Supplementary Table B. Crude and adjusted odds ratios of neonatal mortality and morbidity for Apgar score of 7, 8 and 9 at 1, 5, and 10 minutes among term, singleton live births in Sweden, 1999˗2016** | | | | | | | | |
| --- | --- | --- | --- | --- | --- | --- | --- | --- |
|  | **Apgar score at 1 min** | |  | **Apgar score at 5 min** | |  | **Apgar score at 10 min** | |
|  | **Crude OR (95% CI)** | **Adjusted OR (95% CI)*** |  | **Crude OR (95% CI)** | **Adjusted OR (95% CI)*** |  | **Crude OR (95% CI)** | **Adjusted OR (95% CI)*** |
| **Neonatal mortality** | |  |  |  |  |  |  |  |
| Apgar scores | |  |  |  |  |  |  |  |
| 7 | 3.0 (1.5-6.0) | 2.9 (1.4-6.0) |  | 8.7 (3.2-23.5) | 5.4 (1.3-21.9) |  | 40.1 (16.5-97.4) | 29.8 (9.5-94.0) |
| 8 | 3.3 (1.9-5.8) | 2.7 (1.5-5.0) |  | 7.7 (4.9-11.9) | 7.4 (4.4-12.2) |  | 14.1 (7.7-25.9) | 14.1 (7.2-27.7) |
| 9 | 1.6 (1.0-2.7) | 1.4 (0.8-2.4) |  | 3.2 (2.4-4.2) | 3.4 (2.5-4.6) |  | 5.2 (3.6-7.4) | 4.8 (3.2-7.1) |
| 10 | Reference | Reference |  | Reference | Reference |  | Reference | Reference |
| **Neonatal infections** | |  |  |  |  |  |  |  |
| Apgar scores | |  |  |  |  |  |  |  |
| 7 | 5.0 (4.5-5.4) | 3.9 (3.5-4.3) |  | 8.9 (7.7-10.2) | 8.2 (7.1-9.5) |  | 11.6 (9.1-14.8) | 10.9 (8.4-14.1) |
| 8 | 3.3 (3.1-3.6) | 2.6 (2.4-2.9) |  | 5.6 (5.3-6.1) | 4.8 (4.5-5.2) |  | 9.7 (8.7-10.8) | 8.5 (7.6-9.5) |
| 9 | 1.6 (1.5-1.7) | 1.5 (1.3-1.6) |  | 2.3 (2.2-2.4) | 2.1 (2.0-2.2) |  | 3.7 (3.5-3.9) | 3.3 (3.1-3.5) |
| 10 | Reference | Reference |  | Reference | Reference |  | Reference | Reference |
| **Asphyxia-related complications** | | | | |  |  |  |  |
| Apgar scores | |  |  |  |  |  |  |  |
| 7 | 8.3 (6.3-10.9) | 6.5 (4.8-8.7) |  | 11.4 (7.6-17.0) | 10.2 (6.7-15.6) | | 9.9 (4.4-22.1) | 10.1 (4.5-22.6) |
| 8 | 4.2 (3.2-5.5) | 3.5 (2.6-4.6) |  | 7.6 (6.2-9.3) | 6.1 (4.8-7.6) |  | 14.4 (10.9-18.9) | 11.4 (8.4-15.5) |
| 9 | 1.6 (1.3-2.1) | 1.4 (1.1-1.8) |  | 2.5 (2.2-2.9) | 2.4 (2.1-2.8) |  | 3.5 (2.9-4.3) | 3.2 (2.6-3.9) |
| 10 | Reference | Reference |  | Reference | Reference |  | Reference | Reference |
| **Neonatal hypoglycemia** | |  |  |  |  |  |  |  |
| Apgar scores | |  |  |  |  |  |  |  |
| 7 | 4.1 (3.8-4.3) | 3.2 (3.0-3.5) |  | 5.0 (4.4-5.7) | 4.3 (3.7-4.9) |  | 6.3 (5.0-8.0) | 5.2 (4.0-6.8) |
| 8 | 3.3 (3.1-3.5) | 2.7 (2.5-2.9) |  | 3.6 (3.3-3.8) | 2.9 (2.7-3.1) |  | 4.7 (4.3-5.3) | 3.9 (3.5-4.4) |
| 9 | 1.8 (1.7-1.8) | 1.6 (1.5-1.7) |  | 2.1 (2.0-2.1) | 1.9 (1.8-2.0) |  | 2.6 (2.5-2.8) | 2.3 (2.2-2.5) |
| 10 | Reference | Reference |  | Reference | Reference |  | Reference | Reference |
| **Respiratory distress** | |  |  |  |  |  |  |  |
| Apgar scores | |  |  |  |  |  |  |  |
| 7 | 14.3 (13.3-15.4) | 14.2 (13.1-15.4) |  | 62.7 (58.3-67.6) | 57.6 (53.1-62.4) | | 112.5 (97.9-129.2) | 105.1 (90.5-122.1) |
| 8 | 7.5 (7.0-8.1) | 7.5 (6.9-8.1) |  | 25.8 (24.8-26.9) | 24.0 (22.9-25.0) | | 62.9 (59.3-66.7) | 58.2 (54.6-62.1) |
| 9 | 1.9 (1.8-2.1) | 2.0 (1.9-2.1) |  | 5.4 (5.2-5.5) | 5.2 (5.1-5.4) |  | 13.2 (12.8-13.6) | 12.4 (12.0-12.9) |
| 10 | Reference | Reference |  | Reference | Reference |  | Reference | Reference |

* Adjusted for maternal factors (age at childbirth, parity, country of birth, education, smoking, cohabitation with a partner, height and early pregnancy BMI) and birth characteristics of the infant (sex, gestational age in weeks, and year of birth).

N/A: Odds ratios could not be reliably calculated because of small number of cases.

| **Supplementary Table C. Association between Apgar scores of 7, 8 or 9 at 1, 5 and 10 minutes and neonatal morbidity by mode of delivery among term, singleton live births in Sweden, 1999˗2016** | | | | | | | | |
| --- | --- | --- | --- | --- | --- | --- | --- | --- |
|  | **Non-instrumental vaginal delivery** | | |  | **Elective cesarean section** | | | |
| **Neonatal Morbidity** | **Apgar score at 1 min** | **Apgar score at 5 min** | **Apgar score at 10 min** |  | **Apgar score at 1 min** | **Apgar score at 5 min** | | **Apgar score at 10 min** |
|  | **Adjusted OR (95% CI)** | **Adjusted OR (95% CI)** | **Adjusted OR**  **(95% CI)** |  | **Adjusted OR (95% CI)** | **Adjusted OR (95% CI)** | **Adjusted OR**  **(95% CI)** | |
| **Neonatal infections** | |  |  |  |  |  | |  |
| Apgar scores |  |  |  |  |  |  | |  |
| 7 vs 10 | 6.3 (4.3-9.2) | 10.6 (8.7-12.8) | 15.6 (11.3-21.5) |  | 3.4 (2.3-5.1) | 4.7 (2.5-8.9) | | 5.6 (2.1-15.4) |
| 8 vs 10 | 3.7 (2.6-5.3) | 5.5 (5.0-6.1) | 10.1 (8.7-11.8) |  | 2.1 (1.5-2.9) | 3.8 (2.7-5.2) | | 5.3 (3.2-8.6) |
| 9 vs 10 | 1.4 (1.0-1.9) | 2.1 (2.0-2.2) | 3.2 (3.0-3.5) |  | 1.3 (1.0-1.7) | 1.8 (1.5-2.3) | | 2.5 (1.9-3.3) |
| **Asphyxia-related complications** | | |  |  |  |  | |  |
| Apgar scores |  |  |  |  |  |  | |  |
| 7 vs 10 | 3.4 (3.1-3.8) | 14.2 (8.2-24.7) | 8.0 (2.0-32.2) |  | 5.0 (2.1-11.7) | 9.3 (3.4-25.8) | | 7.6 (1.0-55.4) |
| 8 vs 10 | 2.9 (2.6-3.2) | 6.9 (5.0-9.4) | 12.5 (8.0-19.6) |  | 2.7 (1.2-5.8) | 3.0 (1.3-6.9) | | 11.6 (5.3-25.3) |
| 9 vs 10 | 1.7 (1.6-1.8) | 2.4 (2.0-2.9) | 3.6 (2.8-4.7) |  | 1.5 (0.8-2.6) | 2.0 (1.2-3.2) | | 2.1 (1.0-4.4) |
| **Neonatal hypoglycemia** | |  |  |  |  |  | |  |
| Apgar scores |  |  |  |  |  |  | |  |
| 7 vs 10 | 2.1 (1.9-2.3) | 5.3 (4.3-6.6) | 5.6 (3.7-8.4) |  | 4.2 (3.5-5.0) | 3.3 (2.4-4.6) | | 3.2 (1.8-5.7) |
| 8 vs 10 | 2.0 (1.8-2.1) | 2.9 (2.6-3.2) | 4.1 (3.4-4.9) |  | 3.2 (2.8-3.8) | 2.9 (2.4-3.4) | | 4.0 (3.1-5.1) |
| 9 vs 10 | 1.4 (1.3-1.5) | 1.9 (1.8-1.9) | 2.2 (2.0-2.3) |  | 1.9 (1.7-2.2) | 2.2 (2.0-2.4) | | 2.7 (2.3-3.0) |
| **Respiratory distress** | |  |  |  |  |  | |  |
| Apgar scores |  |  |  |  |  |  | |  |
| 7 vs 10 | 15.3 (13.7-17.1) | 69.2 (61.9-77.4) | 124.8 (101.5-153.4) |  | 19.5 (16.4-23.1) | 34.6 (28.3-42.3) | | 57.7 (40.0-83.2) |
| 8 vs 10 | 7.9 (7.2-8.8) | 25.9 (24.4-27.5) | 71.8 (65.8-78.4) |  | 11.6 (10.0-13.5) | 20.9 (18.8-23.3) | | 40.9 (34.4-48.6) |
| 9 vs 10 | 2.0 (1.8-2.2) | 4.7 (4.5-4.9) | 11.7 (11.1-12.3) |  | 2.8 (2.4-3.2) | 6.3 (5.8-6.8) | | 12.9 (11.8-14.1) |
|  |  |  |  |  |  |  | |  |
|  |  |  |  |  |  |  | |  |
|  |  |  |  |  |  |  | |  |
|  |  |  |  |  |  |  | |  |
|  |  |  |  |  |  |  | |  |
|  |  |  |  |  |  |  | |  |
| **Neonatal Morbidity** | **Emergency cesarean section** | | |  | **Instrumental vaginal delivery** | | | |
|  | **Apgar score at 1 min** | **Apgar score at 5 min** | **Apgar score at 10 min** |  | **Apgar score at 1 min** | **Apgar score at 5 min** | | **Apgar score at 10 min** |
|  | **Adjusted OR (95% CI)** | **Adjusted OR**  **(95% CI)** | **Adjusted OR**  **(95% CI)** |  | **Adjusted OR (95% CI)** | **Adjusted OR (95% CI)** | | **Adjusted OR**  **(95% CI)** |
| **Neonatal infections** | |  |  |  |  |  | |  |
| Apgar scores |  |  |  |  |  |  | |  |
| 7 vs 10 | 3.6 (2.8-4.5) | 4.1 (2.8-6.1) | 4.2 (1.9-9.2) |  | 2.9 (1.7-4.9) | 6.9 (4.8-9.8) | | 8.6 (4.4-16.6) |
| 8 vs 10 | 2.8 (2.3-3.5) | 4.1 (3.4-4.9) | 6.4 (4.9-8.3) |  | 2.0 (1.2-3.4) | 3.2 (2.6-3.8) | | 6.1 (4.7-8.1) |
| 9 vs 10 | 1.6 (1.3-1.9) | 2.2 (2.0-2.5) | 3.2 (2.7-3.7) |  | 1.4 (0.8-2.3) | 1.7 (1.5-1.9) | | 3.0 (2.5-3.5) |
| **Asphyxia-related complications** | |  |  |  |  |  | |  |
| Apgar scores |  |  |  |  |  |  | |  |
| 7 vs 10 | 8.1 (3.7-18.0) | 3.5 (0.9-14.1) | 6.6 (0.9-48.0) |  | 6.3 (0.9-46.1) | 3.4 (0.8-14.1) | | 6.9 (0.9-49.9) |
| 8 vs 10 | 2.7 (1.2-6.2) | 4.8 (2.8-8.5) | 7.6 (3.5-16.4) |  | 3.9 (0.5-28.5) | 4.5 (2.8-7.3) | | 7.2 (3.6-14.2) |
| 9 vs 10 | 2.0 (1.0-4.2) | 2.5 (1.7-3.7) | 2.6 (1.5-4.5) |  | 1.9 (0.3-14.0) | 2.1 (1.5-3.1) | | 1.8 (1.1-3.1) |
| **Neonatal hypoglycemia** | |  |  |  |  |  | |  |
| Apgar scores |  |  |  |  |  |  | |  |
| 7 vs 10 | 3.1 (2.7-3.7) | 2.2 (1.6-3.1) | 2.1 (1.1-4.3) |  | 2.6 (1.7-4.2) | 3.5 (2.4-5.1) | | 10.4 (6.0-18.0) |
| 8 vs 10 | 2.7 (2.4-3.2) | 2.3 (2.0-2.7) | 2.6 (2.0-3.4) |  | 2.3 (1.4-3.5) | 2.4 (2.0-2.8) | | 3.0 (2.2-4.0) |
| 9 vs 10 | 1.7 (1.5-1.9) | 1.9 (1.8-2.1) | 2.1 (1.9-2.4) |  | 1.6 (1.0-2.5) | 1.4 (1.3-1.6) | | 1.9 (1.7-2.2) |
| **Respiratory distress** | |  |  |  |  |  | |  |
| Apgar scores |  |  |  |  |  |  | |  |
| 7 vs 10 | 15.2 (12.4-18.6) | 33.8 (27.7-41.2) | 74.6 (50.1-111.1) |  | 12.6 (6.7-23.5) | 43.1 (34.7-53.6) | | 73.4 (49.5-108.7) |
| 8 vs 10 | 8.5 (7.0-10.4) | 18.5 (16.5-20.7) | 32.2 (27.3-38.0) |  | 5.4 (2.9-10.1) | 16.6 (14.6-18.8) | | 37.8 (32.1-44.6) |
| 9 vs 10 | 2.3 (1.9-2.7) | 6.3 (5.8-6.9) | 12.4 (11.3-13.6) |  | 1.9 (1.0-3.6) | 4.2 (3.7-4.7) | | 10.1 (9.1-11.3) |

† Reference category Apgar score of 10. * Adjusted for maternal factors (age at childbirth, parity, country of birth, education, smoking, cohabitation with a partner, height and early pregnancy BMI) and birth characteristics of the infant (sex, gestational age in weeks, and year of birth).

N/A: Odds rations could not be reliably calculated because of small number of cases.

| **Supplementary Table D. Associations between pregnancy and delivery factors and Apgar scores of 7, 8 or 9 at 1, 5 and 10 minutes among term, singleton live births in Sweden, 1999˗2016** | | | | | | | | |
| --- | --- | --- | --- | --- | --- | --- | --- | --- |
|  | **Apgar score at 1 min** | |  | **Apgar score at 5 min** | |  | **Apgar score at 10 min** | |
| **Risk Factors** | **No.** | **Adjusted OR (95% CI)†** |  | **No.** | **Adjusted OR (95% CI)†** |  | **No.** | **Adjusted OR (95% CI)†** |
| **Gestational diabetes** |  |  |  |  |  |  |  |  |
| Apgar scores |  |  |  |  |  |  |  |  |
| 7 | 692 | 1.1 (1.0-1.2) |  | 51 | 1.4 (1.1-1.9) |  | 9 | 1.1 (0.6-2.2) |
| 8 | 1477 | 1.0 (1.0-1.1) |  | 271 | 1.2 (1.1-1.4) |  | 58 | 1.1 (0.8-1.4) |
| 9 | 11664 | 0.9 (0.8-0.9) |  | 1757 | 1.2 (1.1-1.2) |  | 492 | 1.1 (1.0-1.2) |
| 10 | 1843 | Reference |  | 13597 | Reference |  | 15117 | Reference |
| **Preeclampsia** |  |  |  |  |  |  |  |  |
| Apgar scores |  |  |  |  |  |  |  |  |
| 7 | 1933 | 1.8 (1.7-1.9) |  | 134 | 1.5 (1.2-1.8) |  | 31 | 1.4 (1.0-2.0) |
| 8 | 3901 | 1.6 (1.5-1.7) |  | 800 | 1.4 (1.3-1.6) |  | 189 | 1.4 (1.2-1.6) |
| 9 | 25163 | 1.2 (1.2-1.3) |  | 4374 | 1.3 (1.2-1.3) |  | 1288 | 1.2 (1.1-1.3) |
| 10 | 2614 | Reference |  | 28303 | Reference |  | 32103 | Reference |
| **Chorioamnionitis** | | |  |  |  |  |  |  |
| Apgar scores |  |  |  |  |  |  |  |  |
| 7 | 176 | 3.1 (2.5-4.0) |  | 25 | 4.9 (3.3-7.5) |  | 13 | 9.8 (5.5-17.5) |
| 8 | 299 | 2.3 (1.9-2.9) |  | 103 | 3.7 (3.0-4.5) |  | 38 | 4.9 (3.5-6.8) |
| 9 | 1368 | 1.4 (1.2-1.7) |  | 377 | 2.2 (1.9-2.5) |  | 174 | 3.0 (2.5-3.5) |
| 10 | 119 | Reference |  | 1457 | Reference |  | 1737 | Reference |
| **Placental abruption** |  |  |  |  |  |  |  |  |
| Apgar scores |  |  |  |  |  |  |  |  |
| 7 | 184 | 3.3 (2.7-4.1) |  | 12 | 3.2 (1.8-5.7) |  | 4 | 3.8 (1.4-10.1) |
| 8 | 293 | 2.2 (1.8-2.7) |  | 99 | 3.8 (3.1-4.7) |  | 22 | 2.9 (1.8-4.6) |
| 9 | 1439 | 1.1 (0.9-1.3) |  | 403 | 2.3 (2.0-2.5) |  | 163 | 2.9 (2.4-3.4) |
| 10 | 185 | Reference |  | 1587 | Reference |  | 1912 | Reference |
| **Premature rupture of membranes** | | |  |  |  |  |  |  |
| Apgar scores |  |  |  |  |  |  |  |  |
| 7 | 222 | 1.3 (1.1-1.6) |  | 33 | 1.7 (1.2-2.5) |  | 6 | 1.1 (0.5-2.4) |
| 8 | 541 | 1.4 (1.3-1.7) |  | 124 | 1.1 (0.9-1.4) |  | 32 | 1.1 (0.8-1.6) |
| 9 | 4677 | 1.3 (1.2-1.5) |  | 695 | 1.1 (1.0-1.2) |  | 241 | 1.1 (1.0-1.3) |
| 10 | 496 | Reference |  | 5084 | Reference |  | 5657 | Reference |
| **Induced labour** | | |  |  |  |  |  |  |
| Apgar scores |  |  |  |  |  |  |  |  |
| 7 | 8731 | 1.7 (1.7-1.8) |  | 555 | 1.2 (1.1-1.4) |  | 145 | 1.3 (1.0-1.5) |
| 8 | 19312 | 1.6 (1.6-1.7) |  | 3429 | 1.3 (1.2-1.3) |  | 776 | 1.1 (1.0-1.2) |
| 9 | 145404 | 1.3 (1.3-1.3) |  | 22431 | 1.2 (1.2-1.2) |  | 6825 | 1.3 (1.2-1.3) |
| 10 | 15419 | Reference |  | 162451 | Reference |  | 181120 | Reference |
| **Vaginal instrumental delivery*** | | |  |  |  |  |  |  |
| Apgar scores |  |  |  |  |  |  |  |  |
| 7 | 12468 | 18.1 (17.2-19.0) | | 546 | 3.6 (3.3-4.0) |  | 120 | 2.9 (2.3-3.6) |
| 8 | 22391 | 12.3 (11.7-12.9) | | 4237 | 4.2 (4.0-4.4) |  | 920 | 3.5 (3.2-3.8) |
| 9 | 64822 | 3.4 (3.3-3.6) |  | 18277 | 2.2 (2.2-2.2) |  | 5927 | 2.3 (2.3-2.4) |
| 10 | 2108 | Reference |  | 78729 | Reference |  | 94822 | Reference |
| **Elective cesarean section*** | | |  |  |  |  |  |  |
| Apgar scores |  |  |  |  |  |  |  |  |
| 7 | 2694 | 0.6 (0.5-0.6) |  | 509 | 3.2 (2.8-3.5) |  | 145 | 3.7 (2.9-4.5) |
| 8 | 7143 | 0.6 (0.6-0.6) |  | 2369 | 2.2 (2.1-2.3) |  | 657 | 2.6 (2.4-2.9) |
| 9 | 85080 | 0.6 (0.5-0.6) |  | 11594 | 1.1 (1.1-1.2) |  | 4030 | 1.5 (1.4-1.5) |
| 10 | 20666 | Reference |  | 101111 | Reference |  | 110751 | Reference |
| **Emergency cesarean section*** | | |  |  |  |  |  |  |
| Apgar scores |  |  |  |  |  |  |  |  |
| 7 | 5244 | 1.1 (1.1-1.2) |  | 524 | 2.9 (2.6-3.2) |  | 122 | 2.7 (2.2-3.4) |
| 8 | 10649 | 0.9 (0.9-0.9) |  | 2596 | 2.2 (2.1-2.3) |  | 720 | 2.6 (2.3-2.8) |
| 9 | 77055 | 0.7 (0.7-0.7) |  | 12076 | 1.2 (1.2-1.3) |  | 4408 | 1.6 (1.5-1.7) |
| 10 | 12872 | Reference |  | 90624 | Reference |  | 100570 | Reference |
| **Meconium aspiration** | | |  |  |  |  |  |  |
| Apgar scores |  |  |  |  |  |  |  |  |
| 7 | 216 | 52.2 (26.7-102.0) | | 74 | 106.2 (79.9-141.2) | | 34 | 166.4 (112.9-245.1) |
| 8 | 195 | 20.5 (10.5-40.0) | | 190 | 43.3 (35.3-53.2) | | 107 | 76.9 (60.9-97.3) |
| 9 | 305 | 3.7 (1.9-7.2) |  | 221 | 7.7 (6.3-9.3) |  | 233 | 20.4 (17.1-24.4) |
| 10 | 10 | Reference |  | 241 | Reference |  | 352 | Reference |
| † Reference category Apgar score of 10. * Adjusted for maternal factors (age at childbirth, parity, country of birth, education, smoking, cohabitation with a partner, height and early pregnancy BMI) and birth characteristics of the infant (sex, gestational age in weeks, and year of birth). | | | | | | | | |
| **Reference group spontaneous vaginal delivery | | | | | | | | |
